# CircPCMTD1 Acts as the Sponge of miR-224-5p to Promote Glioma Progression

**DOI:** 10.3389/fonc.2019.00398

**Published:** 2019-05-22

**Authors:** Si-Qi Zheng, Yue Qi, Jun Wu, Fen-Li Zhou, Hao Yu, Lu Li, Bo Yu, Xiao-Fan Chen, Wei Zhang

**Affiliations:** ^1^Shenzhen Key Laboratory for Translational Medicine of Dermatology, Biomedical Research Institute, Shenzhen Peking University-The Hong Kong University of Science and Technology Medical Center, Shenzhen, China; ^2^Department of neurology, Peking University Shenzhen Hospital, Shenzhen, China; ^3^Department of Medicine Laboratory, Peking University Shenzhen Hospital, Shenzhen, China; ^4^Department of Dermatology, Peking University Shenzhen Hospital, Shenzhen, China

**Keywords:** glioma, circPCMTD1, miR-224-5p, oncogene, U118MG cells, U251 cells

## Abstract

Glioma is the most common malignant tumor of the central nervous system with high morbidity and mortality. Circular RNAs (circRNAs) are abundant non-coding RNAs, which contribute to tumor progression by competing with other endogenous RNAs such as microRNA (miRNA). MiRNA are a class of small non-coding RNAs, which interrupt the translation of target mRNAs. CircPCMTD1 (hsa-circ-0001801) is a newly discovered circRNA that was found to be significantly upregulated in glioma. However, its function is unclear. In this study, circPCMTD1 upregulation promoted the cell viability, migration and invasion dramatically, while the inhibition of circPCMTD1 led to a significant reduction of tumor growth *in vivo*. MiRNAs microarray analyses on circPCMTD1 silencing models in U251 and U118MG cells were performed, and the results suggested that circPCMTD1 knockdown could upregulate the expression of miR-224-5p and downregulate the expression of mTOR, one of miR-224-5p targets, in both cell lines. According to the prediction from circular RNA interactome and Targetscan, there was a complementary sequence in circPCMTD1 for miR-224-5p. Dual-luciferase reporter assay demonstrated that circPCMTD1 were targets of miR-224-5p. RIP assay was also performed to further confirm their directly interaction. Overexpression of miR-224-5p inhibited the viability and proliferation, migration, and invasion of U251 and U118MG glioma cells. In conclusion, circPCMTD1 could contribute to the promotion of glioma progression, and it may serve as the sponge of miR-224-5p to exert its function.

## Introduction

Glioma, characterized by its high morbidity and mortality, is the most common malignant tumor of the central nervous system, which accounts for more than 80% of malignant brain tumors worldwide (1, 2). High lethality and aggression of glioma ultimately leads to high recurrence rate after surgery. Despite advances in combination of radiotherapy or chemotherapy, malignant gliomas have an unfavorable prognosis with the overall median survival times (OS) of 37.6 months for anaplastic gliomas, 14.4 months for glioblastoma multiforme (GBM), and 78.1 months for low-grade gliomas (WHO Grade II) according to the Chinese Glioma Genome Atlas (CGGA) statistics (3, 4). Therefore, it is essential to explore the key molecules and mechanisms underlying the pathogenesis and development of glioma, which could potentially identify effective targets for the treatment of glioma.

MicroRNAs (miRNAs) are a class of small non-coding RNAs which interrupt the translation of target mRNAs with miRNA response elements (MRE) (5). MiRNAs participate in a variety of biological processes, such as cell differentiation, proliferation, mobility, and survival (6). In addition, dysregulated miRNAs can act as oncogenes or tumor suppressor genes that play important roles in tumorigenesis and progression of malignancies (7–9). For example, miR-20a and miR-106b have a putatively causative involvement in malignant progression of pediatric brainstem glioma (BSG) and are regarded as possible novel targets for constraining the rapid fatal course of pediatric BSG (10). In addition, there are also some other miRNAs involved in the progression of glioma. Yang et al found that five miRNAs with diagnostic and preventive potentials were significantly correlated with survival time, including hsa-miR-155, hsa-miR-199b, hsa-miR-10a, hsa-miR-1274b, and hsa-miR-455 (11).

Circular RNAs (circRNAs) are endogenous, abundant, non-coding RNAs with important biological functions (12). CircRNAs have a high tolerance to exonucleases because of their special covalent loop structure without 5′ cap and 3′ tail, which may serve as special molecular markers in some diseases including tumors (13–15). In recent years, circRNAs have been proven to be associated with human neurodegenerative diseases (16), cancers (17), as well as the development of human brain (18). CircRNAs could function as microRNA (miRNA) sponges (19–21). For example, ciRS-7 adsorbs miR-7, and is the first characterized circRNA to support the miRNA sponge model (22). Additionally, more and more circRNAs were proven to be miRNA sponges in different cancers, such as colorectal cancer, gastric cancer, breast cancer and glioma (23–25). Recent studies have also shown that circRNAs participated in the progression of tumors through regulating gene expression at transcriptional and post-transcriptional levels (16, 26). Circ-TTBK2 regulated the miR-217/HNF1b/Derlin-1 pathway to promote the progression of glioma (27); and circ-SHKBP1 regulated the angiogenesis of U87 glioma-exposed endothelial cells through miR-544a/FOXP1 and miR-379/FOXP2 pathways (28). In glioma cells, Li et al. reported that upregulated hsa_circ_0007534 could regulate cell growth and migratory capacity via sponging miR-761 (29).

CircPCMTD1 is a circRNA found to be significantly upregulated in glioma tissues (30). CircPCMTD1 continuously increased while circTUBA1B continuously decreased during all differentiation stages in the cardiovascular System (14, 31), but the function of circPCMTD1 remains unclear in glioma. This study was designed to investigate the expression and molecular function of circPCMTD1 in glioma cells, and to explore the molecular mechanisms in aspect of its interaction with miRNAs. Our work would elucidate the important role of circPCMTD1 in glioma and could provide a novel insight into circRNA-targeted treatment in glioma.

## Materials and Methods

### Cell Cultures and Transfection

Glioma cell lines U251 and U118MG, and HEK-293T cells were obtained from American Type Culture Collection (ATCC, Manassas, VA, USA). Cells were cultured in complete growth medium, which is composed of Dulbecco's Modified Eagle's Medium (DMEM) supplemented with 10% Fetal Bovine Serum (FBS, Gibco, USA) and 1% Penicillin/Streptomycin. All cell lines were kept in in a 37°C incubator with 5% CO_2_. U251 and U118MG cells were seeded in six-well plates or 96-well plates. For siRNA transfection of each well, 100 nM siRNA (RiboBio, China) was added with RNA iMAX Reagent (Sigma, USA). For plasmid transfection, plasmids were added with ViaFect™ Transfection Reagent (Promega, USA) according to the manufacturer's guidelines. MiR-224-5p mimic, miR-224-5p inhibitor and their negative controls were purchased from Ribobio (Guangzhou,China).

### Plasmid Construction

The 402bp length sequence of circRNA circPCMTD1 was amplified using 293T cell cDNA as a template, with the primers listed as circPCMTD1-F and circPCMTD1-R (Table S1). EcoR I and BamH I are endonuclease sites for plasmid construction with pLCDH-ciR vector. The primers used for amplifying circPCMTD1-WT are LUC-CIR10873-F and LUC-CIR10873-R. CircPCMTD1 mutant of miR-224-5p binding site, containing G to C and A to T substitutions (synthesized by GeneCreate Biological Engineering, China), was amplified with primers LUC-CIR10873-F and LUC-CIR10873-R. The fragments of circPCMTD1-WT and CircPCMTD1 mutant were cloned into the psiCHECK-2 luciferase vector (Promega, USA) with NotI and XholI endonuclease. The sequences of all constructs were confirmed by DNA sequencing (IGE Biotech, China).

### Quantitative Real-Time PCR (qRT-PCR)

Cytoplasmic and nuclear RNA isolation were performed using Nuclear and Cytoplasmic Protein Extraction Kit (BD, China) with RNase inhibitor from the Goscript RT system (Promega, USA) following the manufacturer's instruction. Total RNA was extracted from cultured cells with Trizol (Life Technologies, USA) according to the manufacturer's protocol. RNA purity was evaluated based on the A260/A280 ratio. cDNAs were prepared by reverse transcription with the Goscript RT system (Promega, USA). Expression of miRNAs was normalized in each sample to U6, while the expression of circPCMTD1 was normalized in each sample to GAPDH. The bulge-loop RT primer, qRT-PCR primers specific for miRNAs, and primers for the back-splice region of circPCMTD1 were synthesized by GeneCreate Biological Engineering (China). Amplification was performed with the SYBR green SuperMix (Bio-Rad,USA) on a CFX96 Touch™ Real-Time PCR Detection System (Bio-Rad,USA). Data were collected and analyzed with the Bio-Rad software using 2^−ΔΔ*ct*^ method for qualification of the relative expression levels of miRNAs and circPCMTD1. All experiments were repeated at least three times. The primers used in this study were listed in Table S1.

### Cell Proliferation Assay

Cells were seeded in a 96-well plate and transfected with 10 nM siRNA. The cell viability was measured every 12 h after transfection with Cell Counting Kit 8 (MCE, China) according to the manufacturer's instructions. The plate was incubated for 3 days. The number of viable cells was evaluated by the absorbance at 450 nm.

### Cell Migration Assay

After scratched with 20 μL pipette tips, the cells were incubated with serum-free medium for 24 h. Image pro plus 6.0 software (Media Cybernetics, USA) was used to measure distance, while GraphPad Prism (GraphPad Software, USA) was used for statistical analysis. For the transwell migration assay, 600 μL media supplemented with 10% FBS was added to the lower chamber (Corning, USA). Cells resuspended in serum-free media were then added to the upper insert after transfection. After incubation for 24 h, Transwell membranes were fixed with 4% Paraformaldehyde fixative (PFA) and stained with 0.5% crystal violet solution for 30 min. Cells stayed in the upper surface of the membrane were wiped off with a cotton swab. The cells adhering to the lower surface of the membrane were photographed under light microscope, counted by Image J and analyzed by GraphPad Prism (GraphPad Software, USA). The numbers of cells counted in five random fields.

### Cell Invasion Assay

Transwell chambers coated with 20 μL Matrigel were used to assess to the invasion ability of glioma cells. Glioma cells resuspended in serum-free media were added to the upper insert after transfection. Transwell membranes were fixed with 4% PFA and stained with 0.5% crystal violet after 48 h. Cells stayed in the upper surface of the membrane were wiped off with a cotton swab. The cells adhering to the lower surface of the membrane were photographed under light microscope, counted by Image J and analyzed by GraphPad Prism (GraphPad Software, USA). The numbers of cells counted in five random fields.

### Flow Cytometry (FCM) Assay

Cells were seeded into a 6-well plate and cultured in standard conditions for 36 h. After transfection, the cells were harvested, fixed with 70% cold ethanol at 4°C overnight and washed with PBS twice. The cells were subjected to PI staining with FxCycle™PI/RNase Staining solution (Invitrogen, USA) and detected by Gallios flow cytometer (Beckman, USA) according to manufacturer's instructions. Cells were trypsinized, resuspended and incubated with 500 μL of PI. Data were collected by FACS Aria flow cytometer (BD Biosciences) at 488 nm, and the cell cycle radio was determined by Modfit software (Verity Software House, USA).

### Nude Mouse Tumorigenicity Assay

Six-week-old male nude mice were purchased from Guangdong animal experimental center and housed in a specific pathogen-free room in the Animal Facility at the Biomedical Research Institute, Shenzhen Peking University–the Hong Kong University of Science and Technology Medical Center. Cells transfected with the siRNA of circPCMTD1 or control siRNA were diluted in Matrigel mix. Mice were injected subcutaneously with 0.1 mL of the suspension into the back flank. After 6 weeks, the mice were killed, and the tumors were dissected and weighed. Tumor volume was calculated by the modified ellipsoid formula: tumor volume (cm^3^) = xy^2^, where x is the greatest longitudinal diameter and y is greatest transverse diameter. This study was performed in accordance with animal use protocols approved by the Committee for the Ethics of Animal Experiments, Shenzhen Peking University-The Hong Kong University of Science and Technology Medical Center (SPHMC) (protocol number 2011–004). All animals were handled in accordance with the guidelines of the Committee for the Ethics of Animal Experiments, SPHMC.

### Dual Luciferase Reporter Assay

HEK-293T cells were seeded in 12-well plates (Costar, USA), which were divided into two mimic groups, two mimic-NC groups, two inhibitor groups, and two inhibitor-NC groups. Each group was transfected with 200 μg psiCHECK-2-PCMTD1-WT or psiCHECK-2-PCMTD1-mut. Reporter vectors were co-transfected with miR-224-5p mimics, miR-224-5p inhibitor and related negative controls by using Viafect reagent (Promega, USA). Luciferase activity was measured with the dual luciferase reporter assay system (Promega, USA) 36 h after transfection. One hundred fifty microliter Lysis Buffer was added to each pore and placed in a new centrifugal tube for 5 min. The mixture was centrifuged for 30 s and placed on ice. In the darkroom, 10 μL lysate and 10 μL Luciferase Substrate were added to the new centrifugal tube and mixed quickly within 3 s. Then 10 μL Stop Reagent was added and mixed quickly within 3 s, and Renilla luciferase activity was detected. Firefly luciferase activity was normalized to renilla luciferase activity. All experiments were repeated at least three times.

### RNA Immunoprecipitation (RIP) Assay

The U251 cells were used to perform these assays with the RIP kit (BersinBio, China). Briefly, more than 5 × 10^7^ cells were collected and washed with PBS, then cross-linked at 37°C for 10 min. Cells were lysed with 1 ml lysis buffer and fully homogenized with a 0.4 mm syringe. After DNA was removed, different biotinylated antisense probes (0.2 nmol) were added to the circRNA-RIP system, and one biotinylated antisense probe (0.2 nmol) targeting the adaptor sequence was added to the circRNA-RIP system. The probes and beads were prepared. The probes were incubated at 65°C for 10 min and hybrided at room temperature for 30 min, then denatured at 50°C for 5 min and hybrided at room temperature for 90~180 min. Six hundred microliter streptavidin-coated magnetic beads was added. Magnetic beads were collected by magnetic frame and transferred to new tube. Non-specifically bound RNAs were removed by washing, and Trizol reagent was used to reverse transcribed miRNAs to cDNA specifically interacting with circRNAs. PCR and qRT-PCR were used to analyze binding strength after reverse transcribing the miRNAs. The probes used in the RIP assay are listed in Table S2.

### Statistical Analysis

The software SPSS 19.0 was used for data analysis. CCK8 assays were assessed using repeated measures ANOVA. For other experimental data, comparisons among multiple groups were performed with one-way ANOVA, followed by LSD's *post hoc* analysis. Comparisons between two groups were achieved with Student's unpaired *t*-test. The data were presented as mean ± s.d from at least three separated experiments. *P* < 0.05 was considered statistically significant.

## Results

### CircPCMTD1 Promoted Cellular Viability and Proliferation

**Figure 1:**
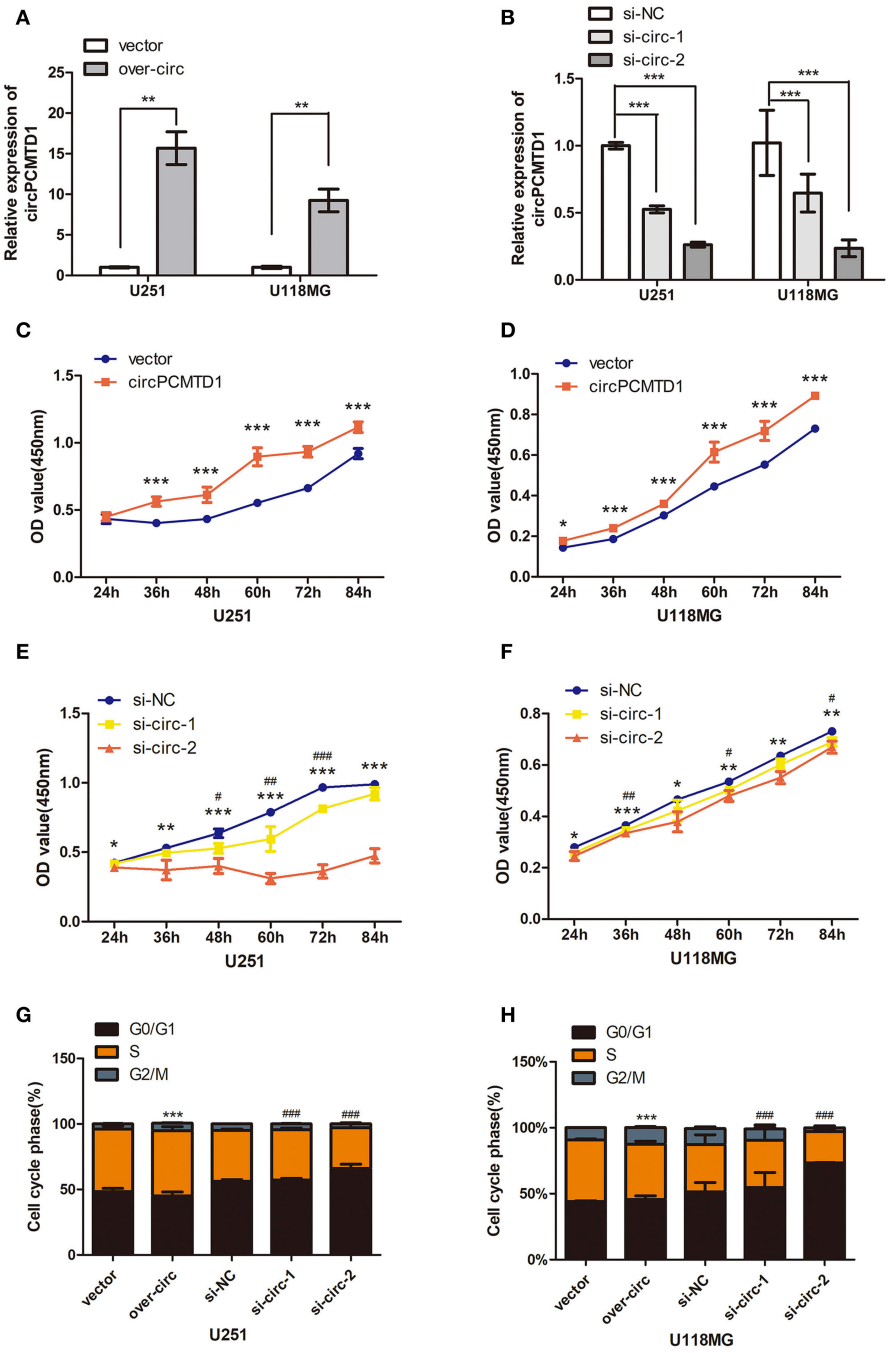
Effect of circPCMTD1 on cells growth and proliferation. **(A)** After transfection of overexpressing vector, the relative expression of circPCMTD1 in U251 and U118MG was detected by qRT-PCR. GAPDH was used as control. Data represent means ± SD (*n* = 3, each group). **(B)** After transfection of circPCMTD1 siRNA and control siRNA, the knockdown efficiency of circPCMTD1 was confirmed by qRT-PCR. GAPDH was used as control. **(C,D)** CCK-8 assays showed that overexpressing circPCMTD1 in the U251 and U118MG cell line, the cell viability was upregulated. **(E,F)**. CCK-8 assays showed that cell proliferation was suppressed when circPCMTD1was silenced. ###*p* < 0.001 or ***p < 0.001 vs. control vector group for si-circ, **P < 0.01 or ##*p* < 0.01 vs. control vector group for si-circ, *P < 0.05 or #*p* < 0.05 vs. control vector group for si-circ, # for si-circ-1 and * for si-circ-2, *n* = 4. **(G,H)**. The cells were accelerated to S/G2/M phase when circPCMTD1 was upregulated. Conversely, U251 and U118MG cells with circPCMTD1 stably silencing exhibited a massive G1 phase arrest. ***p < 0.001 vs. control vector group for over-circ, ###*p* < 0.001 vs. control vector group for si-circ, *n* = 4.

In the previous study, it was shown that the expression level of circPCMTD1 was significantly upregulated in glioma tissues, which suggested that the circPCMTD1 may act as an oncogene in glioma (30). CircPCMTD1 knockdown models and overexpression models were constructed in U251 and U118MG cells, to explore the oncogenic function of circPCMTD1. After transfection of overexpression vector and siRNA, the overexpression and knockdown efficiency of circPCMTD1 were confirmed by qRT-PCR (Figures 1A,B). The cell cycle and the cell proliferation rate in these cells were tested. It was found that circPCMTD1 overexpression significantly improved the cell viability and proliferation of U251 and U118MG cells (Figures 1C,D), while inhibition of circPCMTD1 reduced their cell viability and proliferation (Figures 1E,F).

In addition, the cell cycle was accelerated to S/G2/M phase when the expression of circPCMTD1 was upregulated (Figures 1G,H) in both U251 and U118MG cells, while massive G1 phase arrest was observed when the expression of circPCMTD1 was stably silenced (Figures 1G,H). However, knockdown of circPCMTD1, by two junction-specific siRNAs in U251 and U118MG cells, significantly decreased cell cycle acceleration and cell viability (Figures 1E–H). It indicated that silencing circPCMTD1 could induce cell cycle arrest and reduce proliferation in glioma cells.

### Effects of circPCMTD1 on Cellular Migration and Invasion

**Figure 2:**
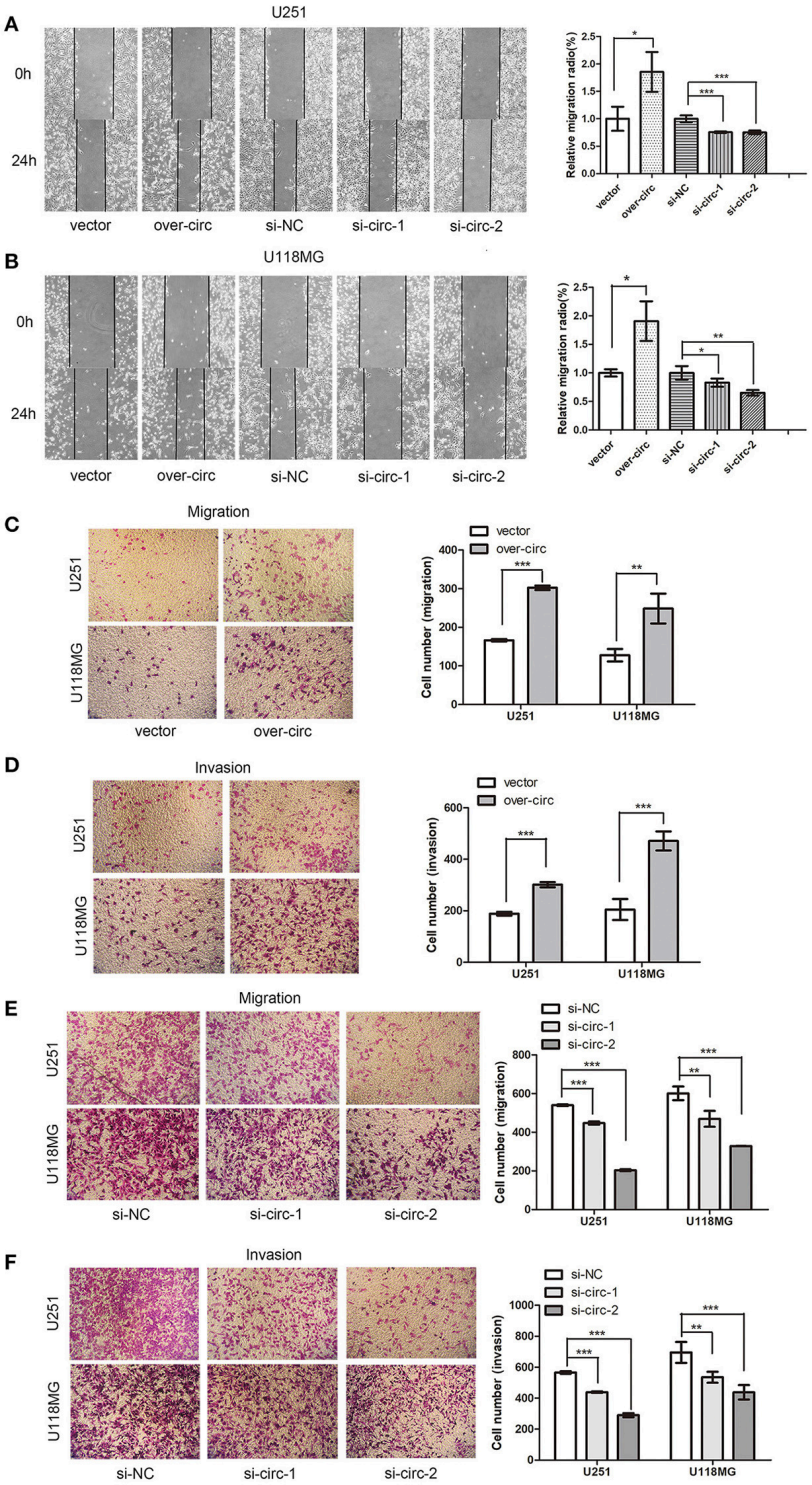
Overexpression of circPCMTD1 promoted cell migration and invasion, while knockdown of cells inhibited cell migration and invasion. **(A)** Wound-healing assays showed that circPCMTD1 overexpression promoted migration of U251 cells, and silencing circPCMTD1 suppressed the cell migration. Statistical analysis of three independent experiments was performed. **(B)** Wound-healing assays showed that circPCMTD1 overexpression promoted migration of U118MG cells, and silencing circPCMTD1 suppressed the cell migration. Statistical analysis of three independent experiments was performed. **(C)** Transwell analysis showed circPCMTD1 overexpression promoted the migration of U251 and U118MG cells. Statistical analysis of three independent experiments was shown in panel. **(D)** Transwell analysis showed circPCMTD1 overexpression promoted the invasion of U251 and U118MG cells, Statistical analysis of three independent experiments was shown in panel. **(E)** Transwell analysis showed circPCMTD1 knockdown promoted the invasion of U251 cells. Statistical analysis of three independent experiments was shown in panel. **(F)** Transwell analysis showed the invasion was suppressed after circPCMTD1 knockdown in U118MG cells. Statistical analysis of three independent experiments was shown in panel. Data were expressed as mean ± SD. *P < 0.05, **P < 0.01 represents statistical difference, ***P < 0.001 represents statistical difference.

The wound-healing assays showed that circPCMTD1 overexpression enhanced the migration ability of U118MG and U251 cells. When circPCMTD1 was downregulated, the migration ability was suppressed significantly, especially by the si-circ-2 siRNA in U118MG cells (Figures 2A,B). Transwell migration assays also suggested that migration ability and invasion ability of both U118MG and U251 cells was enhanced after overexpression of circPCMTD1 (Figures 2C,D), while the silencing model inhibited the migration and invasion of glioma cells (Figures 2E,F). It suggested that circPCMTD1 may serve as an oncogene in glioma cells.

### Knockdown of circPCMTD1 Inhibited Glioma Cells Growth *in vivo*

**Figure 3:**
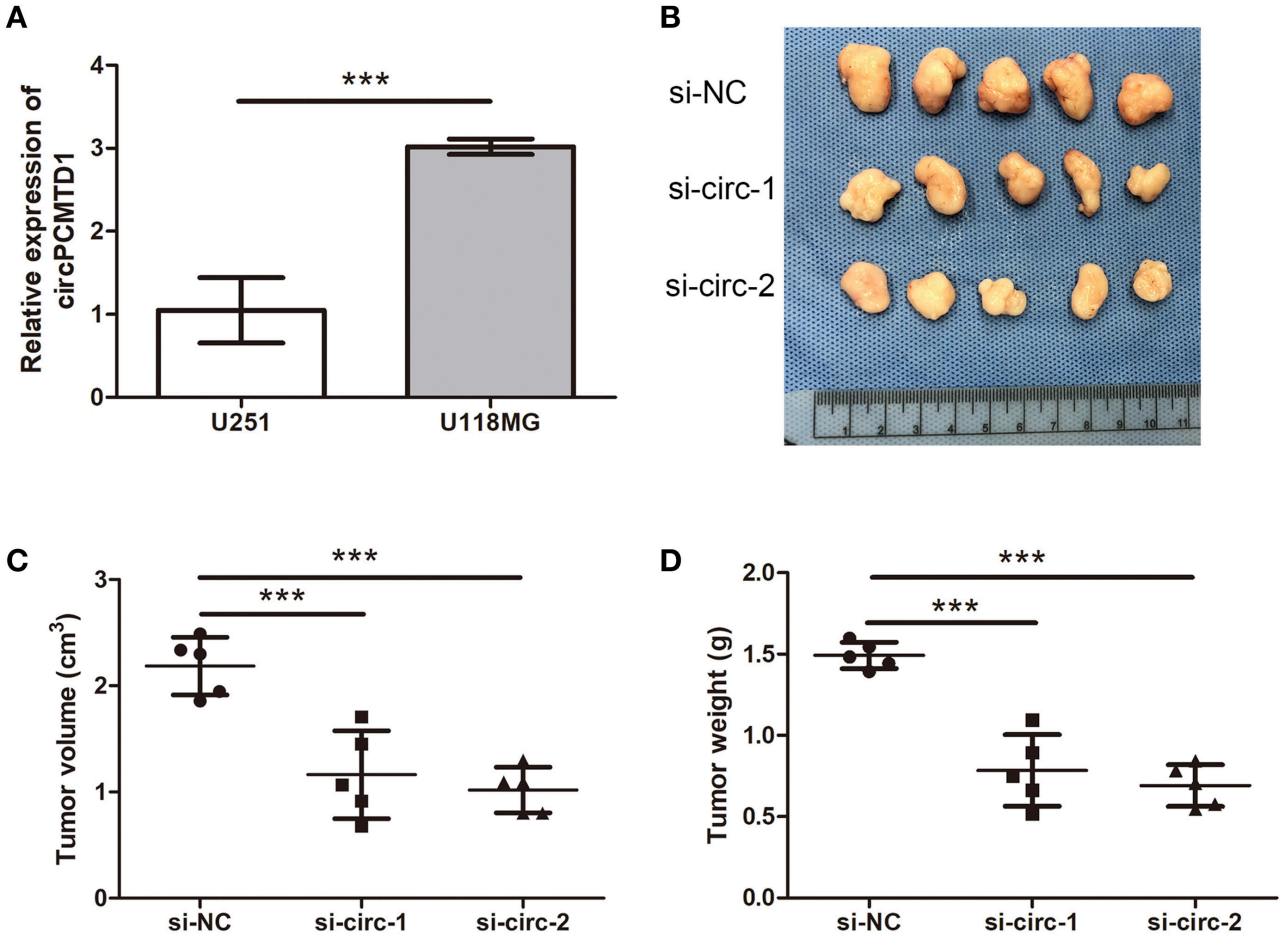
Knockdown of circPCMTD1 inhibited cells growth *in vivo*. **(A)**The relative expression level of circPCMTD1 in U251 and U118MG cells were detected by qRT-PCR. The U118MG cells had higher expression levels of circPCMTD1. **(B)** Representative images of xenografts tumor (five mice per group) in nude mice. Tumors from nude mice after injection of U118MG cells transfected with si-NC, si-circ-1 or si-circ-2. **(C)** Tumor volume was monitored from each group after 35 d. **(D)** Tumor weights were measured from each group after 35 d. All the data are shown as the mean ± S.D., ***P < 0.001.

The expression level of circPCMTD1 in different glioma cells was analyzed. It is a significantly higher level of circPCMTD1 in U118MG cells (Figure 3A). Previous results suggested that downregulation of circPCMTD1 may inhibit the progression of tumor. To further investigate the function of circPCMTD1 in tumor growth and invasion *in vivo*, xenograft experiments were performed. It was found that inhibition of circPCMTD1 led to a significant reduction of tumor growth *in vivo* (Figures 3B–D).

### MiRNAs Profiles of circPCMTD1 Knockdown Models in Glioma Cells

**Figure 4:**
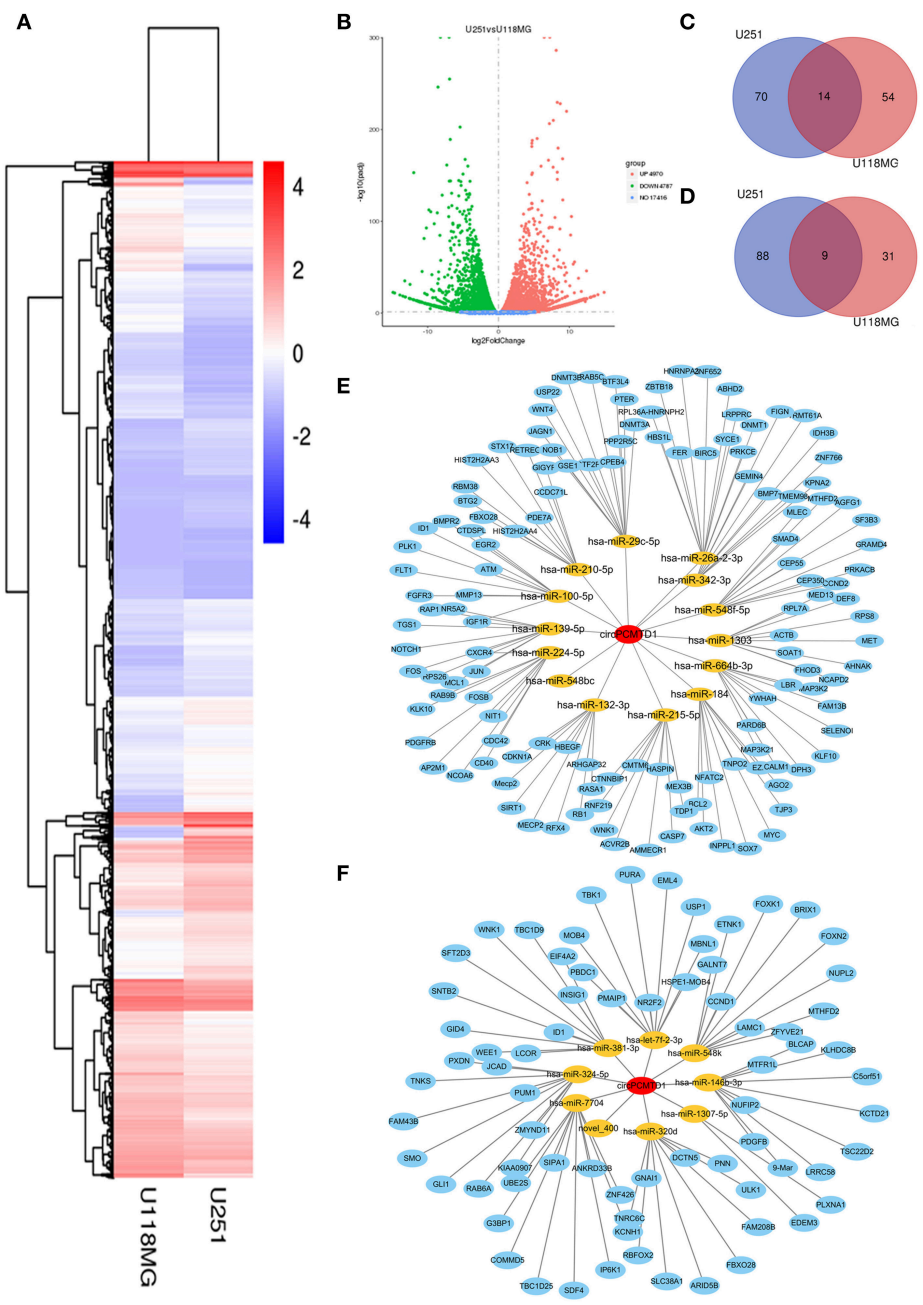
MiRNA expression analysis in knocking-down model of U251 and U118MG cells. **(A)** Hierarchical clustering showed the top upregulated and downregulated miRNAs clusters in knockdown models. **(B)** The variation of miRNAs expression both in U251 and U118MG cells were revealed in the scatter plot. **(C)** The Venn diagram showed the upregulated miRNAs in circPCMTD1 silencing U251 and U118MG cells. **(D)** The Venn diagram showed the downregulated miRNAs in circPCMTD1 silencing U251 and U118MG cells. **(E)** CircRNA-miRNA-mRNA network of upregulated miRNAs in U251 and U118MG cells. **(F)** CircRNA-miRNA-mRNA network of downregulated miRNAs. The miRNAs surrounding the central point circPCMTD1 were in orange and the target genes of each miRNAs were in blue.

**Figure 5:**
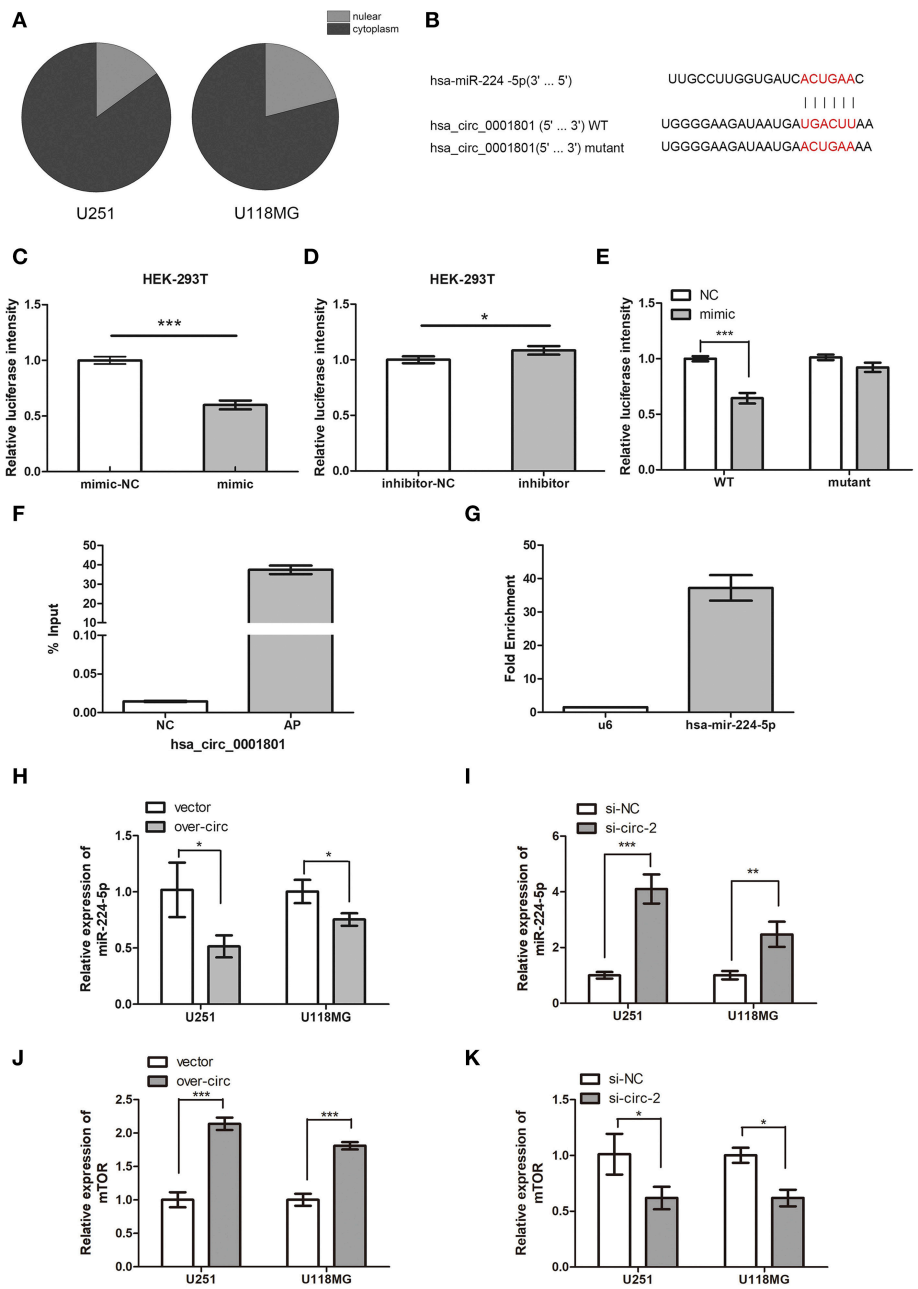
CircPCMTD1 served as the sponge of miR-224-5p. **(A)** The relative expression of circPCMTD1 in glioma cell lines were detected by qRT-PCR after nuclear and cytoplasmic isolation experiments. **(B)** The predicted binding sites of miR-224-5p within circPCMTD1 were shown, and the mutant of this site was shown. **(C)** Luciferase assay of cells cotransfected with miR-224-5p and luciferase reporter containing circPCMTD1. **(D)** Luciferase assay of cells co-transfected with miR-224-5p inhibitors and luciferase reporter containing circPCMTD1. **(E)** The activity of circPCMTD1 sequence reporter containing wild-type or mutant target sites was determined in luciferase assay following co-transfection with miR-224-5p mimics. **(F)** The enrichment efficiency of circPCMTD1 specific probe was detected by qRT-PCR. **(G)**The enrichment fold of miR-224-5p on circPCMTD1 RIP distributed was detected by qRT-PCR. **(H)** The relative expression of miR-224-5p when overexpressing the circPCMTD1 in U251 and U118MG cells was detected by qRT-PCR. **(I)** The relative expression of miR-224-5p when silencing the circPCMTD1 in U251 and U118MG cells were detected by qRT-PCR. **(J)** When circPCMTD1 was overexpressed in U251 and U118MG cells, the relative expression of mTOR was detected by qRT-PCR. **(K)** When the circPCMTD1 was silenced in U251 and U118MG cells, the relative expression of mTOR was detected by qRT-PCR. *P < 0.05, **P < 0.01, ***P < 0.001.

To explore the potential involved miRNAs, miRNA microarray analyses were performed on circPCMTD1 silencing models in U251 and U118MG cells. Hierarchical clustering showed the top upregulated and downregulated clusters of miRNAs in glioma cells when circPCMTD1 was silenced (Figure 4A). The variation of miRNAs expression was revealed in the scatter plot (Figure 4B). Results from the bioinformatics analysis found that circPCMTD1 knockdown could, respectively, affect miRNAs in both cell models (Figures 4C,D). We found 84 miRNAs upregulated with fold change >1.5 in model of U251 cells, while 68 miRNAs were upregulated by the same cutoff in model of U118MG cells, and 14 miRNAs were upregulated in both models (Figure 4C). We also found 97 miRNAs downregulated with fold change >1.5 in model of U251 cells, while 40 miRNAs downregulated by the same cutoff in model of U118MG cells, and 9 miRNAs were upregulated in both models (Figure 4D). The analysis of the circRNA-miRNA-mRNA network were identified and indicated by the bioinformatics analysis (Figures 4E,F), and the miR-224-5p was the only gene to be associated with circPCMTD1 with a complementary sequence in circPCMTD1 (Figure 5B). Therefore, we hypothesize that miR-224-5p may be regulated by circPCMTD1 in glioma cells. All raw data can be found at the NCBI SRA with accession numbers SRR8934915, SRR8934916, SRR8934917, SRR8934918.

### CircPCMTD1 Served as a Sponge of miR-224-5p

To investigate the relationship between circPCMTD1 and miR-225-5p, we firstly identified the intracellular location of circPCMTD1 in glioma cell lines. Nuclear and cytoplasmic fractions were separated from cells and the levels of the nuclear control U6 and cytoplasmic control GAPDH were detected by qRT-PCR, respectively. The results revealed that circPCMTD1 mostly distributed in the cytoplasm of glioma cells (Figure 5A), indicating the possibility of its role as the molecular sponge to interact with miRNA, which led to the liberation of corresponding miRNA-targeted transcripts (32). According to the prediction from circular RNA interactome and Targetscan, a complementary sequence in circPCMTD1 for miR-224-5p was identified (Figure 5B). Thus, a dual-luciferase reporter assay was then performed to confirm the interaction between circPCMTD1 and miR-224-5p. The results showed that transfection of a miR-224-5p mimic significantly attenuated the activity of luciferase reporter containing wild-type circPCMTD1 compared with the mimic control (Figure 5C), while miR-224-5p did not affect the activity of luciferase reporter containing a mutant circPCMTD1 fragment (Figure 5E). Transfection of miR-224-5p inhibitor slightly reduced the activity of the luciferase reporter containing wild-type circPCMTD1 (Figure 5D). It suggested that circPCMTD1 can bind miR-224-5p in the cytoplasm in baseline expression.

To explore the direct interaction between miR-224-5p and circPCMTD1 in baseline levels of their expression, RIP assays were performed. U251 cells were used to investigate the binding interaction at natural level. Figures 5F,G showed that miR-224-5p was significantly enriched in RNAs retrieved from the circPCMTD1 complex, indicating the specific interaction between circPCMTD1 and miR-224-5p in glioma cells. In addition, overexpression of circPCMTD1 in U251 and U118MG can reduce the expression levels of miR-224-5p (Figure 5H), while upregulation of miR-224-5p was observed when circPCMTD1 was silenced in glioma cells (Figure 5I). Our results revealed that circPCMTD1 could serve as a sponge of miR-224-5P in glioma cells.

The results of luciferase assays indicated that mTOR was a direct target of miR-224-5p, which is consistent with a previous study of miR-224-5p in a gastric cancer cell (Figure S1) (33). Dysregulation of mTOR was found in different types of tumors including glioblastoma (34, 35). Downregulation of mTOR was also observed when circPCMTD1 was silenced, while upregulation of mTOR was also observed when circPCMTD1 was overexpressed in glioma cells (Figures 5J,K).

### Effects of miR-224-5p on the Cell Viability and Proliferation

**Figure 6:**
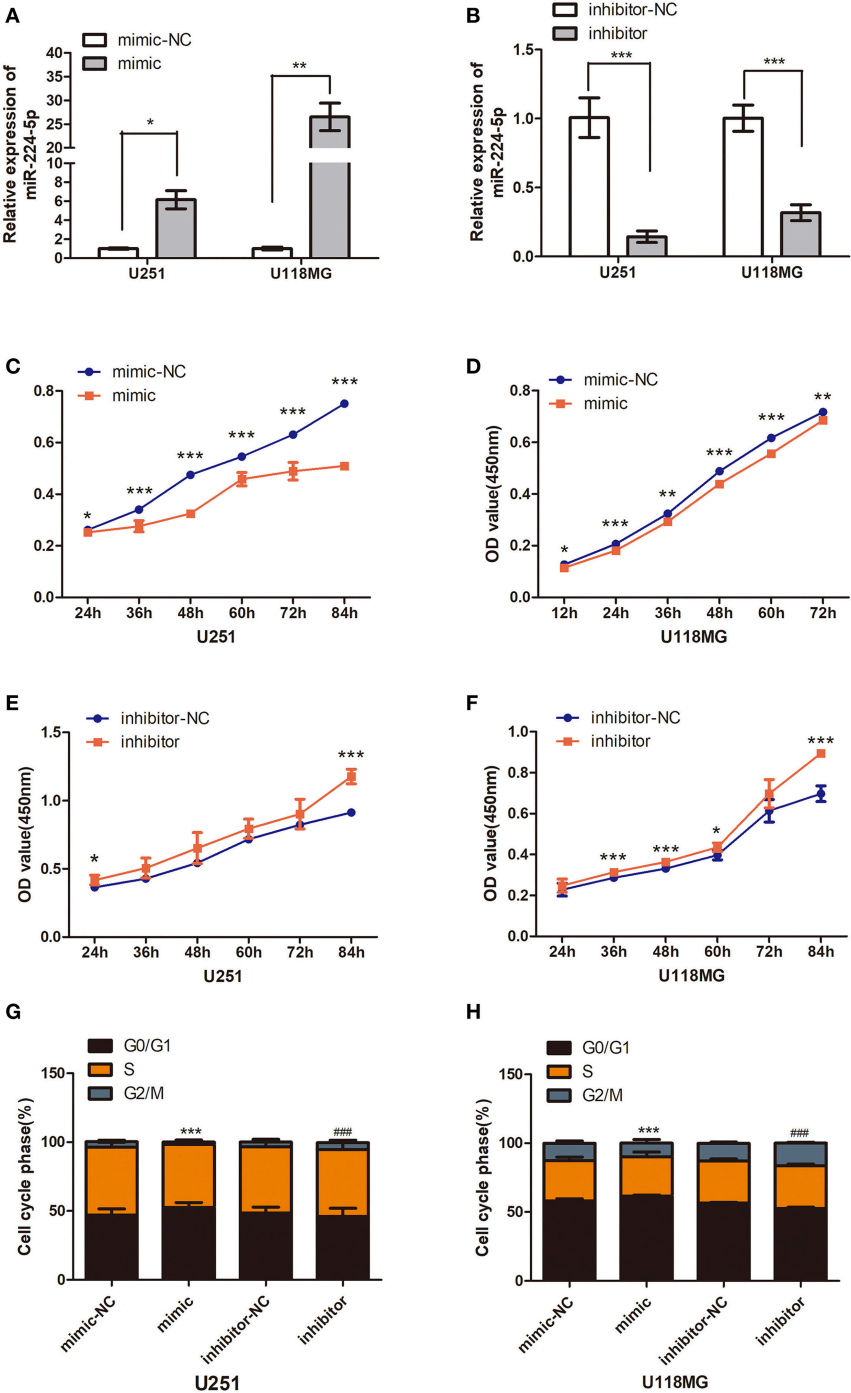
Effect of miR-224-5p on cells growth and proliferation. **(A)** The overexpressing efficiency of mir-224-5p in U251 and U118MG cells was detected by qRT-PCR. **(B)** The silencing efficiency of miR-224-5p in U251 and U118MG cellswas detected by qRT-PCR. **(C,D)** CCK8 assaysshowed overexpressing the miR-224-5p suppressed the cell viability in the U251 and U118MG cell line from 24 to 84 h. **(E,F)** CCK8 assays showed silencing the miR-224-5p upregulated the cell viability in the U251 and U118MG cell line from 24 to 84 h. ***p < 0.001, **P < 0.01, *P < 0.05, *n* = 4. **(G,H)** 24 h after transfection, the cell cycle was arrested to S/G2/M phase when upregulated the expression of miR-224-5p. Conversely, miR-224-5p stably silencing in U251 and U118MG cells exhibited a massive G1 phase upregulated compared with their control cells. t ***p < 0.001 vs. control group for mimic-NC, ###*p* < 0.001 vs. control group for inhibitor-NC, *n* = 4.

Overexpression or silencing of miR-224-5p was performed to further understand their role in the progression of glioma. The transfection expression efficiency was evaluated by qRT-PCR (Figures 6A,B). Overexpression of miR-224-5p inhibited the viability and proliferation of glioma cells U251 and U118MG (Figures 6C,E), while downregulation of miR-224-5p promoted their viability and proliferation (Figures 6D,F). The cell cycle was accelerated to S/G2/M phase when inhibited the expression of miR-224-5p (Figures 6G,H), conversely, miR-224-5p stably overexpressing in U251 and U118MG cells exhibited a massive G1 phase arrest compared with their control cells (Figures 6G,H).

### Effects of miR-224-5p on Cellular Migration and Invasion

**Figure 7:**
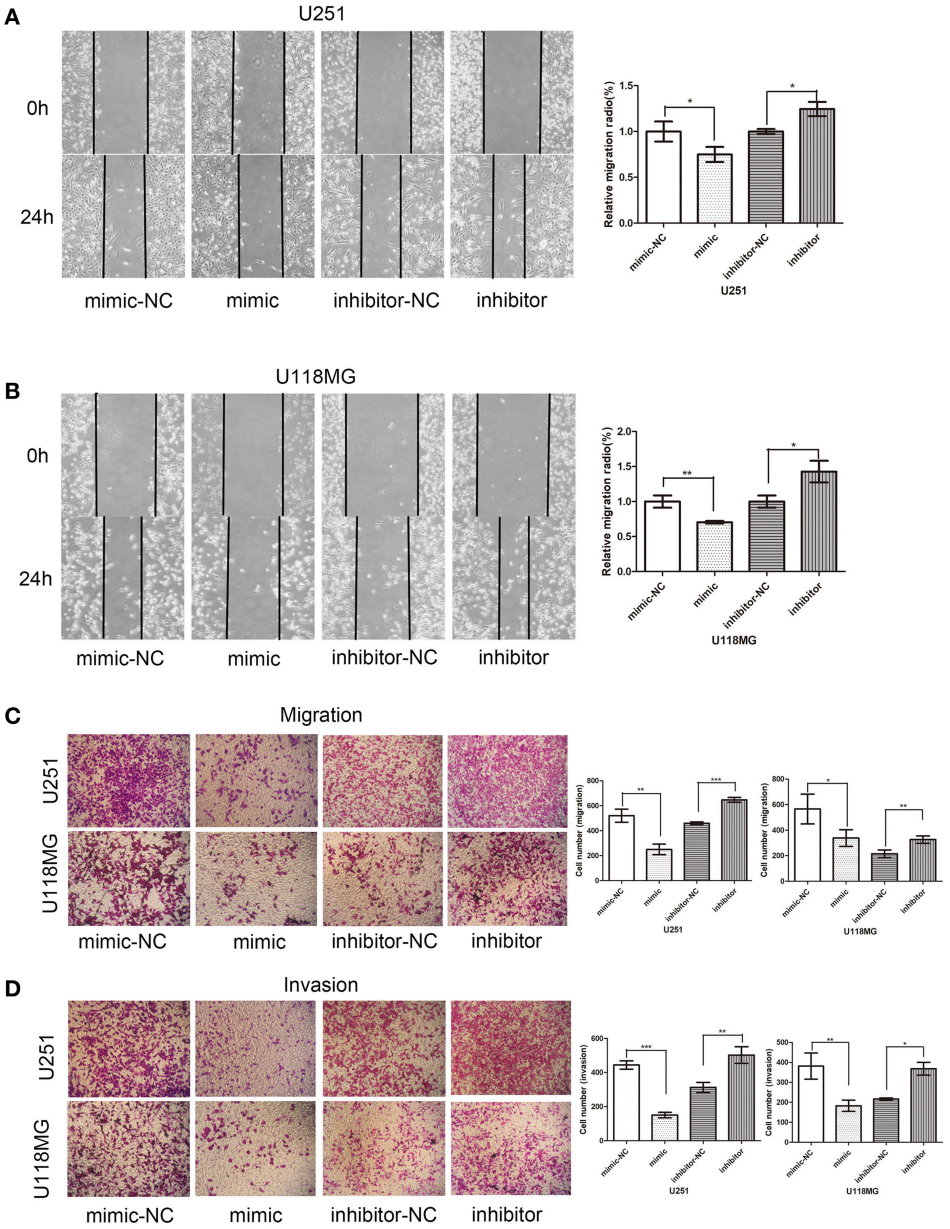
Effect of miR-224-5p on cell invasion and migration. Overexpression of miR-224-5p suppressed cell migration and invasion, while knockdown of miR-224-5p promoted cell migration and invasion. **(A)** Wound-healing assays showed miR-224-5p overexpression suppressed migration of U251 cells. **(B)** Wound-healing assays showed miR-224-5p overexpression suppressed migration of U118MG cells. **(C)** Transwell analysis showed miR-224-5p overexpression suppressed migration of U251 and U118MG cells. Conversely, migration was promoted when miR-224-5p was downregulated. **(D)** Transwell with Matrigel analysis showed miR-224-5p overexpression suppressed invasion of U251 and U118MG cells. Conversely, invasion was promoted when miR-224-5p was downregulated. Statistical analysis of three independent experiments was shown in panel. *P < 0.05, **P < 0.01, ***p < 0.001.

The effects of miR-224-5p on cell migration and invasion were explored through wound-healing assays and transwell assays. In order to confirm that circPCMTD1 promoted glioma progression though its sponge activity of miR-224-5p, miR-224-5p inhibitor was used to examine whether the tumor-suppressing effect of circPCMTD1 silencing could be blocked by miR-224-5p. In the wound-healing assays, we found that the migration ability of U118MG and U251 was inhibited by overexpression of miR-224-5p (Figure 7). Transwell migration assays suggested that both U118MG and U251 cells migration was enhanced after inhibition of miR-224-5p. Both silencing of circPCMTD1 or upregulation of miR-224-5p significantly reduced the cell migration and invasion. All these findings suggested that miR-224-5p inhibited the cell migration and invasion in glioma cells.

The mammalian Target of Rapamycin (mTOR) signaling network could regulate several physiologic and pathologic cellular processes, such as cell growth, proliferation, and survival, which is very important in tumor pathogenesis (36). Silencing of mTOR, as a target of miR-224-5p, could inhibit the viability, proliferation, migration, and invasion in U251 and U118MG cells, which has similar effects with silencing of circPCMTD1 and overexpression of miR-224-5p (Figure S1). It suggested that circPCMTD1 regulates mTOR expression and affects the glioma cell phenotype by competing with miR-224-5p.

## Discussion

CircRNAs are a new class of long non-coding RNAs that play a role in gene expression regulation. Recent studies have shown that circRNAs participate in the progression of tumors (9) and other diseases (2). The curative effects on glioma by surgery and/or radiotherapy and chemotherapy have remained limited due to the complicated mechanisms of glioma, which led to poor prognosis and high mortality. Although the mechanism in the pathogenesis of glioma remained undetermined, accumulating evidence suggested that a large number of circRNAs and miRNAs were involved in the pathogenesis of glioma. Previous studies have revealed that circRNAs could modulate gene expression by acting as miRNAs sponges in various tumors (1, 9, 37). For instance, hsa_circ_0007534 promoted the progression of glioma through the miR-761/ZIC5 regulatory loop (29). To determine whether 20 circRNAs are functionally associated with pluripotency, Yu et al first examined their temporal expression pattern during embryoid body differentiation. RT-qPCR analyses showed that there was a downregulated trend of circPCMTD1 in the human embryonic stem cells stage (38). However, the function of circPCMTD1 in glioma has not been investigated. In the present study, the circPCMTD1 silencing can significantly inhibit glioma progression *in vivo* and *in vitro*. Importantly, luciferase reporter assay was induced to reveal the underlying mechanisms of circPCMTD1. CircPCMTD1 was demonstrated to be the oncogene of glioma cells for the first time, which may serve as a potential therapy target for glioma.

According to the World Health Organization (WHO) guidelines (39), neuropathologists classified the tumors as grades II–IV. U251 and U118MG cell lines are derived from different grades of brain tumors. U118MG, a WHO grade IV human GBM cell line, derived from highly malignant GBM tumors. U251 glioma cells are derived from a grade II neuroblastoma (39–41). The morphology of U251 cells are epithelial, while the morphology of U118MG cells are mixed and both glioblastoma and astrocytoma cells are present (42, 43). It was found that the U251 cells had a different migration speed when compared to U118MG cells. The U251 cells closed the wound area at a rate of 10%/day, while the U118MG cells invaded the wound area at a rate of about 20–25%/day (34). These may be the cause of their different behaviors relating to cell viability, proliferation, migration and invasion in our experiments. Upregulated circPCMTD1 in U251 cells had a more significant promotion of cell viability and proliferation than in U118MG cells, because lower baseline expression of circPCMTD1 in U251 cells may make the cell more sensitive.

MicroRNAs participated in the regulation of tumorigenesis (11). MiR-224-5p was upregulated in circPCMTD1 knockdown glioma cells. CircPCMTD1 could directly bind miR-224-5p and had an effect on miR-224-5p expression levels. The expression level of miR-224-5p was decreased in the circPCMTD1 overexpressed glioma cells. Several studies have revealed a decreased level of miR-224-5p in osteogenic differentiation in different cells (44, 45). It was found that miR-224-5p was able to distinguish tumors from non-neoplastic penile tissues with high sensitivity and specificity. It was shown that miR-224-5p expression level was downregulated in uveal melanoma cell lines and tissues. FTH1P3 expression was inversely correlated with the miR-224-5p expression in uveal melanoma tissues. Investigation of the underlying mechanism may be a target for the treatment of uveal melanoma (46). Analysis of the Cancer Genome Atlas data showed lower miR-224 expression levels and higher expression levels of its target API5 in male glioblastoma multiforme patients were correlated with poorer survival. In addition, they also found that overexpression of miR-224 could increase the radiation sensitivity of glioblastoma cells, which appeared as promising options for the treatment of glioblastoma (47). It suggested that the miR-224-5p may serve as a potential target for glioma therapy. In addition, inhibition of mTOR could suppress glioma cells progression, and mTOR has an interaction with miR-224-5p. In conclusion, circPCMTD1 was demonstrated to be an important oncogene involved in the progression of glioma.

## Ethics Statement

This study was carried out in accordance with the recommendations of the guidelines of the Committee for the Ethics of Animal Experiments, Shenzhen Peking University-The Hong Kong University of Science and Technology Medical Center. The protocol was approved by the animal use protocols approved by the Committee for the Ethics of Animal Experiments, Shenzhen Peking University-The Hong Kong University of Science and Technology Medical Center.

## Author Contributions

S-QZ carried out the molecular and cellular studies, participated in the animal experiments and drafted the manuscript. HY, LL, and YQ performed the statistical analysis. JW, BY, and F-LZ participated in this design. X-FC revised the manuscripts and the statistical analysis. WZ conceived of the study and helped to draft the manuscripts. All authors read and approved the final manuscripts.

### Conflict of Interest Statement

The authors declare that the research was conducted in the absence of any commercial or financial relationships that could be construed as a potential conflict of interest.
