# Supplementary material for: CircPCMTD1 Acts as the Sponge of miR-224-5p to Promote Glioma Progression
Source: Front Oncol. 2019 May 22;9:398. doi: 10.3389/fonc.2019.00398 (PMC6538694; doi:10.3389/fonc.2019.00398)
Supplement: Supplementary file 1 [file Table_1.DOC]

**Supplementary Data**

**Table S1 List of** oligonucleotides used in this study.

| **Primer name** | **Sequence（5’-3’）** |
| --- | --- |
| **Primers used for RT-qPCR** | |
| CircPCMTD1-QPCR-F | CTGAAGTTATGGAAGCATTG |
| CircPCMTD1-QPCR-R | ATGGCTTCCAATATTGCACTTG |
| h-GAPDH-F | GATGAGAAGTATGACAACAGCCT |
| h-GAPDH-R | AGTCCTTCCACGATACCAAAGT |
| U6-F | GCTTCGGCAGCACATATACTAAAT |
| U6-R | CGCTTCACGAATTTGCGTCTCAT |
| Bulge-loop-RT | CTCAACTGGTGTCGTGGAGTCGGCAATTCAGTT  GAGCTAAACGG |
| miR-224-QPCR-F | ACACTCCAGCTGGGTCAAGTCACTAGTGGTTCC |
| miR-224-QPCR-R | TGGTGTCGTGGAGTCG |
| mTOR-QPCR-F | TTTGGGGACTGCTTTGAGGTT |
| mTOR-QPCR-R | CCAGTTCAGCAAGGGGTCATA |
| **Primers used for plasmid Construction** | |
| CircPCMTD1-F | CGGAATTCTGAAATATGCTATCTTACAGGATTAATTTATTTTTGGAAATC |
| CircPCMTD1-R | CGGGATCCTCAAGAAAAAATATATTCACCTAAAATTAAGCCCACCATTG |
| LUC-CIR10873-F | AATTCTAGGCGATCGCTCGAGGATTAATTTATTTTTGGAAATCAAGTGC |
| LUC-CIR10873-R | ATTTTATTGCGGCCAGGCGGCCGCCTAAAATTAAGCCCACCATTGTACTT |

**Table S**2 The probes used in the RIP assay

| **Name** | **Probe sequence（5’-3’）** |
| --- | --- |
| hsa_circ_0001801 | TTGCACTTGATTTCCAAAAATAAATTAATCCCTAAAATTAAGCCCACCATTGTACTTAAA |
| Lac Z | CAAACGGCGGATTGACCGTAATGGGATAGGTCACGTTGGTGTAGATGGGCGCATCGTAAC |
| Lac Z | CACCACATACAGGCCGTAGCGGTCGCACAGCGTGTACCACAGCGGATGGTTCGGATAATG |
| Lac Z | CCAATCCGCGCCGGATGCGGTGTATCGCTCGCCACTTCAACATCAACGGTAATCGCCATT |

**Figure S1.** Effects of mTOR on cell viability, migration, and invasion.

**(A)** The predicted binding sites of miR-224-5p in the region of the human mTOR 3'-UTR were shown. **(B)** The direct modulation of miR-224-5p on the 3'-UTR of the mTOR were shown by luciferase reporter assay. **(C)** The knockdown efficiency of mTOR siRNA in U251 and U118MG was detected by qRT-PCR. GAPDH was used as control. Data represent means ± SD (n = 3, each group). **(D)** CCK-8 assays showed that after mTOR was silenced in the U251 cells, the cell viability was suppressed significantly. **(E)** CCK-8 assays showed that silencing of mTOR in the U118MG cells inhibited cell viability. ***p < 0.001 versus control vector group for si-mTOR, **P <0.01 versus control vector group for si-mTOR, *P <0.05 versus control vector group for si-mTOR, n=4. **(F)** Wound-healing assays showed mTOR silencing suppressed migration of U251 and U118MG cells. **(G)** Statistical analysis of Wound-healing assays. **(H)** Transwell analysis showed downregulation of mTOR suppressed migration of U251 and U118MG cells. **(I)** Statistical analysis of cell migration by transwell assays. **(J)** Transwell with Matrigel analysis showed silencing mTOR suppressed invasion of U251 and U118MG cells. **(K)** Statistical analysis of cell migration by transwell assays. Statistical analysis of three independent experiments was shown in panel.
