# Supplementary figures and images for: CircPCMTD1 Acts as the Sponge of miR-224-5p to Promote Glioma Progression
Source: Front Oncol. 2019 May 22;9:398. doi: 10.3389/fonc.2019.00398 (PMC6538694; doi:10.3389/fonc.2019.00398)

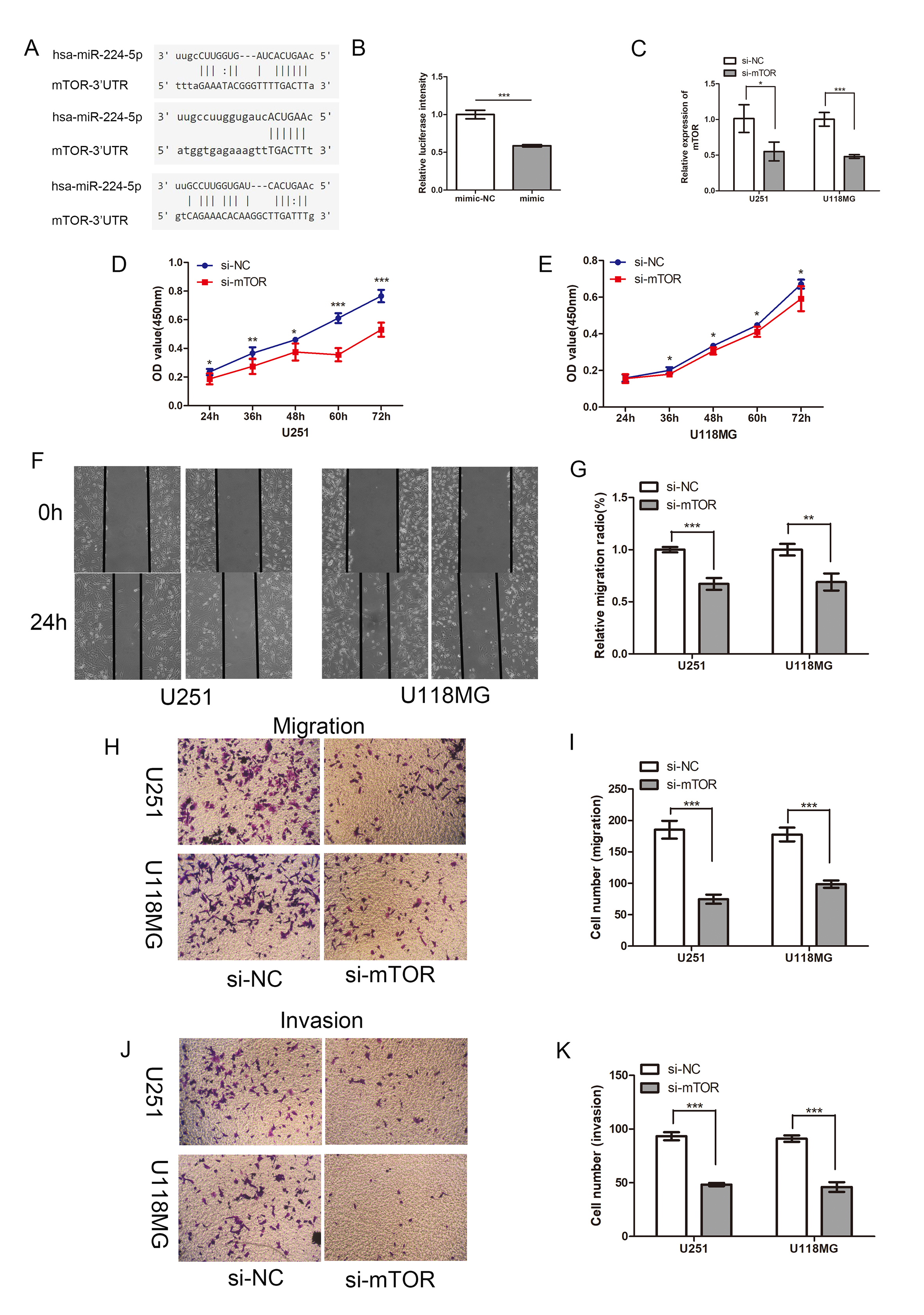

Supplement: Supplementary file 2 [file Image_1.JPEG]
